# Case Report: CD19 and CD20 monoclonal antibodies with sequential chemotherapy for refractory acute B-lymphocytic leukemia in children

**DOI:** 10.3389/fimmu.2023.1280759

**Published:** 2023-11-17

**Authors:** Jia-Nan Li, Yu Li, Ji-Xun Lin, Li-Na Wang, Xiao-Li Zhang, Juan Ouyang, Du-Bo Chen, Shao-Qian Chen, Jun-Xun Li, Xue-Qun Luo, Yan-Lai Tang, Li-Bin Huang

**Affiliations:** ^1^ Department of Pediatrics, The First Affiliated Hospital, Sun Yat-sen University, Guangzhou, China; ^2^ Department of Clinical Laboratory, The First Affiliated Hospital, Sun Yat-sen University, Guangzhou, China

**Keywords:** blinatumomab, refractory acute B-lymphocytic leukemia, allo hematopoietic stem cell transplantation, rituximab, minimal residual disease

## Abstract

**Objective:**

This paper observes the efficacy of chemotherapy combined with CD19 and CD20 monoclonal antibodies in clearing minimal residual disease (MRD) and bridging transplantation for refractory acute B-lymphoblastic leukemia (B-ALL) in children and reviews the literature.

**Methods:**

A 4-year-old boy diagnosed with B-ALL in our hospital was treated with the SCCLG-ALL-2016 protocol. MRD and gene quantification decreased after induction but remained persistently positive, with poor efficacy. After this patient received three cycles of consolidation chemotherapy combined with blinatumomab and rituximab, MRD and fusion gene quantification became negative, and he received allogeneic hematopoietic stem cell transplantation (allo-HSCT).

**Results:**

During the use of monoclonal antibodies, neurotoxicity, CRS, or other side effects did not occur. Before transplantation, MRD became negative, and the bone marrow had been in complete remission since transplantation (13 months).

**Conclusion:**

Chemotherapy combined with blinatumomab for refractory B-ALL in children can bring a better remission rate for patients and is a means of bridging transplantation. Nevertheless, sequential CD20 monoclonal antibody therapy is the first report , and no adverse effects were observed in our case. It is well tolerated and can be used as one of the treatments for refractory B-ALL.

## Introduction

Acute lymphoblastic leukemia (ALL) is a common malignancy in childhood. B type accounts for 75%-85% of cases with good outcomes in children and adolescents, but 15-20% of patients still present with refractory or relapsed disease (1), with long-term survival rates of only 50% after relapse and relatively poor outcomes (2). Thus, better and new treatment strategies with high efficacy are needed. Immunotherapy combined with conventional chemotherapy has been a hot topic in relapsed/refractory ALL (R/R ALL) in recent years. Among them, the FDA has approved blinatumomab to treat R/R ALL (3), and it has shown promising results in clearing MRD and improving remission rates in the first clinical real-world study in children (4). The NCCN guidelines also suggested that blinatumomab can be used in pediatric R/R B-ALL (5). Therefore, this paper reports a case of refractory pediatric B-ALL, that was treated by chemotherapy combined with blinatumomab and rituximab. After the MRD of bone marrow turned negative, the patient suffered allo-HSCT and has sustained complete remission to date. We also review the relevant medical literature.

## Definition

Relapse is defined as the reappearance of primitive cells (>5%) in the peripheral blood or bone marrow or any extramedullary fraction after reaching CR. Based on the latest NCCN 2023 guideline, we defined refractory as a state in which the bone marrow does not reach CR or MRD remains higher than 1% after two standard inductions.

## Materials and methods

A 4-year-old boy was admitted to our hospital on November 23, 2021, with fever and pallor. On examination, no superficial lymph nodes were palpable, the liver was 0.5 cm below the ribs and soft, and the spleen was not palpable. His blood count showed 11.2×10^9^/L leukocytes, 2.06×10^9^/L neutrophils, 68 g/L hemoglobin, 31×10^9^/L platelets, 67% primitive and naive lymphocytes on bone marrow smear, 62.7% abnormal naive B-lymphocytes using flow cytometry (FCM), and immunophenotyping suggesting expression of HLA-DR, CD10, CD19, CD20, CD22, and CD18, CD10, CD19, CD20, CD22, CD34, CD38, CD79a. IgVH(+), TCRβ(+), TCRγ(+). The chromosomal karyotype is 46,XY. Whole exon sequencing and RNA-Seq showed that PAX5-AUTS2 gene fusion was positive, and next-generation sequencing (NGS) showed 66.49% IGH-Sequence A significant clones. In addition, the child was currently untreated, and he had no relevant past medical history or family history of similar conditions. Based on the above, he was diagnosed with acute B-lymphocytic leukemia (ALL-BIV). Since the diagnosis of this patient was made, we started the SCCLG-ALL-2016 protocol, and the trial is registered with the Chinese Clinical Trial Registry (Chi-CTR; https://www.chictr.org.cn/; number ChiCTR2000030357). Based on this patient’s subsequent bone marrow condition, we also used a combination of CD19 and CD20 monoclonal antibodies and received allo-HSCT.

## Results

**Figure 1:**
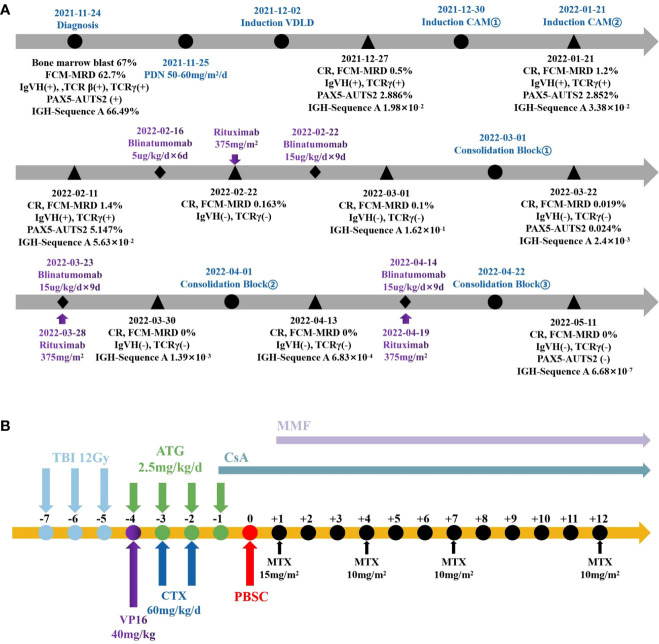
The Schema of therapy in this patient. **(A)** Pre-transplant protocol. **(B)** Process of pretreatment and transplantation.

**Figure 2:**
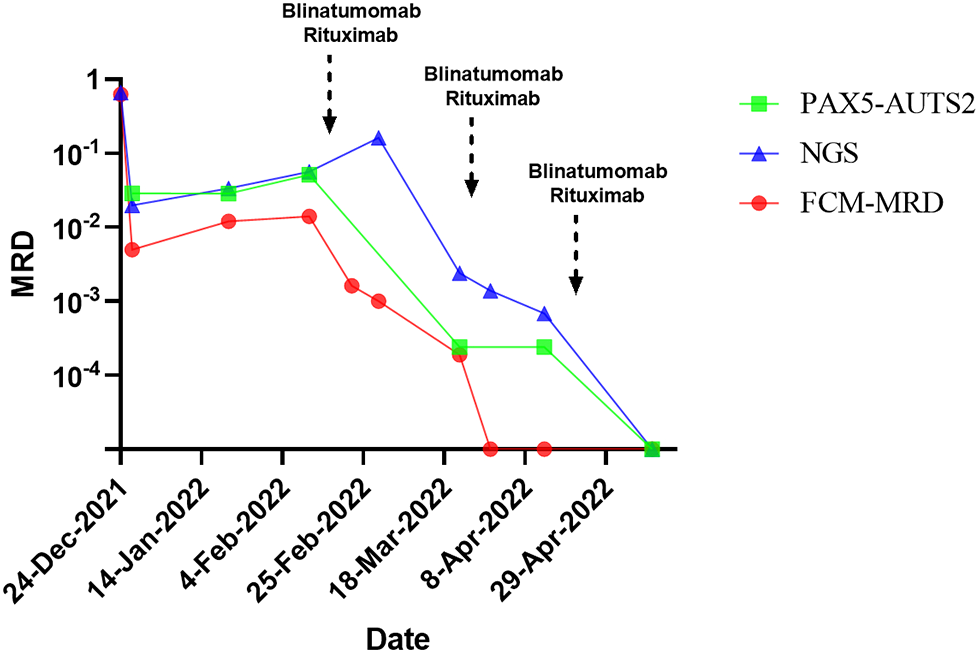
The time of antibodies addition and trend of PAX5-AUTS2 gene quantification, NGS and flow.

The prednisone test started on November 25, 2021 (**Figure 1A**), and the results showed sensitivity. The induction of VDLD started on December 2, 2021, and d15 MRD still showed 22.9% phenotypically abnormal naive B lymphocytes, while d33 MRD monitoring by FCM (FCM-MRD) showed 0.5%. Other results showed IgVH(+), TCRγ(+), and 2.886% PAX5-AUTS2 fusion gene quantification, and NGS showed 1.98×10^2^. The evaluation of prognosis was divided into a high-risk group. After two rounds of induction of CAM, the bone marrow smear did not show any abnormalities, but the FCM-MRD still showed 1.4% abnormal naive cells and 5.147% PAX5-AUTS2 fusion gene, increasing than before. Therefore, we started using blinatumomab on February 2, 2022. An initial dose of 5ug/m^2^/d was escalated after six days to 15ug/m^2^/d for nine days. Before infusion, we added dexamethasone as an anti-inflammatory and antiallergic agent. In addition, we added rituximab on the sixth day of blinatumomab application. After the blood picture rebounded, we added consolidation of Block One. After this course of combination treatment, bone marrow showed 0.019% FCM-MRD and 0.024% PAX5-AUTS2 fusion gene, which was significantly lower than before (**Figure 2**). Therefore, we added blinatumomab in combination with rituximab during each period of consolidation therapy, and the courses of treatment and dosage are shown in the figure. After the third consolidation of Block in combination with blinatumomab and rituximab, the bone marrow smear, FCM-MRD, fusion gene quantification, Ig/TCR, and Ig NGS quantification returned to normal. No adverse effects such as fever, CNS toxicity, or liver function abnormalities, were observed during treatment with monoclonal antibodies. The patient started pretreatment on May 17, 2022 and was treated using allo-HSCT from an unrelated donor on May 5, 2022 (**Figure 1B**), with granulocyte and platelet engrafted on day+11. During HSCT, peri-implantation syndrome, graft-versus-host disease (GVHD, skin grade 1), BK virus-associated cystitis, AKI1 stage 1, infectious fever, upper respiratory tract infection, myelosuppression after radiotherapy, hypoproteinemia, abnormal liver function, hypomagnesemia, and hypokalemia occurred, which were cured eventually, and then in order to prevent relapse, we used decitabine several times. The child is now in continuous complete remission with 99.32% to 99.72% bone marrow chimerism. Written and verbal informed consent was obtained from the patient for this study.

## Discussion

Blinatumomab is a bispecific T-cell binding antibody structure that mediates the formation of synapses between T cells and target cells, one of which recognizes tumor-expressed antigens and the other recognizes CD3 in T cells (6), and it has shown significant efficacy and a low incidence of adverse events in R/R ALL in children. The first study of blinatumomab in children occurred in 2016 (4), which established a 28-day course of blinatumomab at 15ug/m^2^ as the maximum tolerated dose, and the results of the phase II study suggested that 27 (39%) of the final 70 patients achieved CR and 14 (52%) patients had negative MRD, indicating that despite multiple high-risk features at baseline, blinatumomab provided rapid and profound remission with low rates of severe cytokine release syndrome (CRS) and neurotoxic reactions. In the RIALTO trial (7), an expanded international study, the enrolled population consisted of pediatric B-ALL patients with CD19-positive B-cell precursor acute lymphoblastic leukemia (BCP-ALL) in second or later relapse, any relapse after allo-HSCT, or refractory to other treatments. The results showed that among patients with ≥5% blasts at baseline, 59% (59/98) achieved CR within the first two cycles, and 79% (46/98) achieved MRD response. For those with <5% blasts, the MRD response rate was 92% (11/12). The median relapse-free survival (RFS) for patients with CR in the first two cycles was 8.5 months, validating the efficacy of blinatumomab as salvage therapy in pediatric patients. In children with high-risk first relapse B ALL, a study reported that the application of one course of blinatumomab as consolidation chemotherapy before transplantation resulted in higher MRD remission rates than usual chemotherapy (8), the proportion of which was 90% (44/49) versus 54% (26/48), and a lower incidence of adverse events, suggesting that blinatumomab brings a higher remission rate, depth of remission and better safety for patients compared to chemotherapy, making it more likely for patients to receive subsequent allo-HSCT, further achieving prolonged EFS, OS, and DFS. We also selected pediatric cases treated with blinatumomab with HSCT (**Table 1**) (9–15). However, the premise of blinatumomab as pretransplant transitional chemotherapy is to minimize tumor load, and in a meta-analysis, it was noted that patients with low primary cell levels achieved higher remission rates than those with high primary cells (16), suggesting that chemotherapy should be administered to reduce tumor load prior to blinatumomab treatment in refractory relapsed ALL patients. Although reports suggest one course of continuous infusion for four weeks, our regimen adopted an economic strategy of continuing infusion at a loading dose of 15ug/m^2^ for nine days as a course after six days of dose climbing and then combined with consolidation chemotherapy after hematologic recovery. The above three cycles were repeated, and after MRD became negative, we performed HSCT, obtaining complete remission of bone marrow to date. The drug also has some adverse effects, but the incidence is low compared with conventional chemotherapy. The most frequent complications are hematocrit and fever (4, 17, 18), and the most serious adverse effects are CRS and neurological toxicity (17), but the incidence of grade ≥3 CRS is not high (18). In this case, the child did not develop CRS during use of blinatumomab, and cytokine levels were normal. Otherwise, there were no adverse reactions, such as fever and drowsiness, and the infusion process was smooth, which may be related to the step-climbing treatment we used and the anti-inflammatory and anti-allergic application of dexamethasone before infusion. Moreover, bone marrow remission before blinatumomab treatment was closely related to the incidence of adverse reactions during treatment, and the greater the tumor load was, the higher the incidence of CRS. For such patients, stepwise dosing and prephase treatment such as dexamethasone, are measures to prevent CRS (19).

**Table 1:** Selected pediatric cases treated with blinatumomab with HSCT(Literature review).

Trial reference	Number of patients	Disease status before the initiation of blinatumomab	Cycle of blinatumomab	Outcome
(9)	23	Relapse/Refractory	1-2	23 CR
(10)	4	Refractory	1	2 CR, 2 Dead
(11)	39	Relapse/Refractory	1-9	16 Dead
(12)	9	Relapse/Refractory	1-2	3 CR, 6 Dead
(13)	13	Relapse/Refractory/with severe infection	1-4	10 CR, 3 Dead
(14)	14	Refractory	1-2	8 CR, 2 Dead 4 Relapse
(15)	3	Relapse	1	3 CR
Present case	1	Refractory	3	1 CR

HSCT, hematopoietic stem cell transplantation.

**Figure 3:**
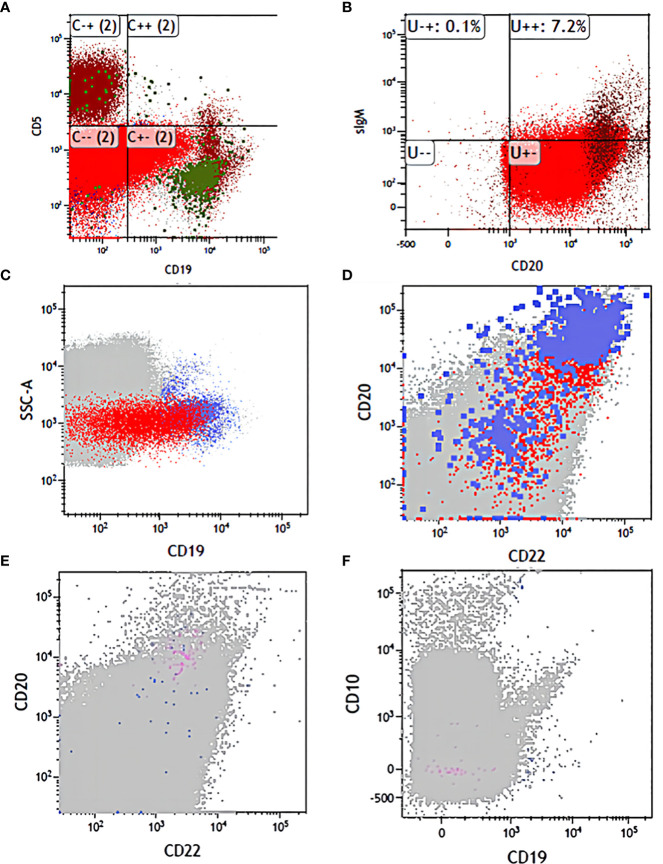
The flow changes before and after treatment. **(A)** CD19 and CD5 expression in diagnosis. **(B)** CD20 and sIgM expression in diagnosis. **(C)** CD19 expression before application of antibodies. **(D)** CD22 and CD20 expression before application of antibodies. **(E)** CD20 and CD22 expression before transplantation. **(F)** CD19 and CD10 expression before transplantation.

However, in fact, such treatment is still ineffective in some patients. Some studies have indicated that the relationship between bispecific antibodies and therapeutic effects is closely related to the function of T cells present in the patients themselves (20). The mechanism of blinatumomab is to form a lysis synapse between T cells and tumor cells, which leads to lysis and apoptosis of tumor cells and promotes T-cell proliferation (6). Therefore, it is worth noting that we treat with blinatumomab first when T cells are not depleted because the monoclonal antibody requires the presence of normal T cells, and if the blood picture has not recovered and T cells are depleted, the monoclonal antibody cannot be sufficiently effective, so our order is that monoclonal antibody used first and consolidation chemotherapy came second. In addition, we should monitor the expression of CD19 molecules of FCM-MRD to avoid poor therapeutic response due to the loss of the target antigen CD19. In this study, CD molecules were monitored during the intervals of chemotherapy to ensure that blinatumomab had sufficient targets to be effective (**Figures 3A–F**). For such patients who do not respond to blinatumomab, combination therapy with other antibodies is expected to overcome the problem of CD19 escape (17), such as inotuzumab ozogamicin, which is still in clinical trials without real-world data. In this case, when we rechecked the FCM-MRD after a creeping dose of blinatumomab, although it was lower than before, it was still positive and showed a high percentage of CD20. Therefore, we added CD20 monoclonal antibody on days 5-6, and after one course of treatment, the FCM-MRD and the percentage of CD19 and CD20 decreased compared with before (**Table 2**), suggesting that this combination regimen was effective in controlling the disease of this child. Therefore, we used this method before consolidation chemotherapy, but due to the economic problems of the child, we did not use the full 28-day course as recommended in the guidelines, but the effect of this dual monoclonal antibody combination is also very promising.

**Table 2:** Expression level of CD19 and CD20 on the blasts at diagnosis and during the treatments.

Date of bone marrow puncture	Phase of treatment	Percentage of CD19	Percentage of CD20
November 24, 2021	diagnosis	20.3%	70.6%
February 11, 2022	post-induction CAM②	24.5%	57.3%
February 22, 2022	post-initial dosage of Blinatumomab	2.1%	47.2%
March 1, 2022	post-creeping dosage of Blinatumomab together with Rituximab	0.2%	0.6%
March 22, 2022	post-consolidation Block①	18.2%	2.6%
March 30, 2022	post-Blinatumomab together with Rituximab	0%	0%

In terms of transplantation, studies have reported significantly better survival and a significantly lower risk of relapse and treatment-related mortality (TRM) in pediatric high-risk ALL patients who received TBI combined with etoposide pretreatment before HSCT compared to those who received clearly myeloablative chemotherapy pretreatment (21). Therefore, with myeloablative conditioning with total body irradiation (TBI, 12Gy, day -7, -6, -5), VP16 (40mg/kg, day -4), CTX (60mg/kg/d, day -3, -2), and ATG (2.5mg/kg/d, day -3, -2, -1), the patient was treated using allo-HSCT from an unrelated donor on May 24, 2022, receiving 7.8×106/kg CD34 cells). Granulocyte and platelet engraftment occurred on day+11, and peripheral blood chimerism suggested 99.76% on day+14. During transplantation, adverse reactions such as peri-implantation syndrome and GVHD occurred during transplantation, which were cured after treatment with methylprednisolone, cyclosporine, MMF, hydration and alkalinization of urine, and cidofovir antiviral therapy, and the bone marrow was in complete remission at follow-up. Certainly, the success of transplantation is based on the premise that dual sequentially monoclonal antibodies combined with chemotherapy clearing MRD, but further studies are needed to determine whether blinatumomab can directly replace chemotherapy in R/R ALL.

## Conclusions

Chemotherapy combined with dual monoclonal antibodies to clear MRD followed up by bridging transplantation is an alternative option for R/R ALL, with few adverse effects and being tolerable during application in this patient, with complete bone marrow remission to date, gaining objective results and improving survival outcomes.

## Data availability statement

The original contributions presented in the study are included in the article. Further inquiries can be directed to the corresponding authors.

## Ethics statement

The studies involving humans were approved by Ethics Committee of the First Affiliated Hospital of Sun Yat-sen University. The studies were conducted in accordance with the local legislation and institutional requirements. Written informed consent for participation in this study was provided by the participants’ legal guardians/next of kin. Written informed consent was obtained from the individual(s), and minor(s)’ legal guardian/next of kin, for the publication of any potentially identifiable images or data included in this article.

## Author contributions

J-NL: Writing – original draft. YL: Writing – original draft. J-XL: Writing – review & editing. L-NW: Writing – review & editing. X-LZ: Writing – review & editing. JO: Software, Writing – review & editing. D-BC: Software, Writing – review & editing. S-QC: Software, Writing – review & editing. JL: Software, Writing – review & editing. X-QL: Writing – review & editing. Y-LT: Writing – review & editing. L-BH: Writing – review & editing.
